# Mitigation behavior prior to COVID-19 vaccination availability is associated with COVID-19 infection and time to vaccination

**DOI:** 10.1371/journal.pone.0283381

**Published:** 2023-03-24

**Authors:** Coralei E. Neighbors, Richard Sloane, Carl F. Pieper, Douglas Wixted, Christopher W. Woods, L. Kristin Newby

**Affiliations:** 1 Hubert-Yeargan Center for Global Health, Duke University, Durham, North Carolina, United States of America; 2 Center for the Study of Aging and Human Development, Duke University Medical Center, Durham, North Carolina, United States of America; 3 Department of Biostatistics and Bioinformatics, Duke University Medical Center, Durham, North Carolina, United States of America; 4 Duke Clinical and Translational Science Institute, Duke University, Durham, North Carolina, United States of America; 5 Departments of Medicine and Pathology, Duke University Medical Center, Durham, North Carolina, United States of America; 6 Duke Clinical Research Institute, Duke University, Durham, North Carolina, United States of America; 7 Division of Cardiology, Department of Medicine, Duke University Medical Center, Durham, North Carolina, United States of America; Uniformed Services University: Uniformed Services University of the Health Sciences, UNITED STATES

## Abstract

**Background:**

Mitigation behaviors reduce the incidence of COVID-19 infection. Determining characteristics of groups defined by mitigation behaviors compliance may be useful to inform targeted public health policies and interventions. This study aimed to identify groups of individuals according to self-reported compliance with COVID-19 mitigation behaviors, define compliance class characteristics, and explore associations between compliance classes and important study and public health outcomes.

**Methods and findings:**

We studied 1,410 participants in the Cabarrus County COVID-19 Prevalence and Immunity longitudinal cohort study (June 2020 to December 2021) who were asked 10 questions regarding compliance with recommended COVID-19 mitigation behaviors. By Latent Class Analysis, 1,381 participants were categorized into 3 classes (most [49.4%], moderately [45.0%], and least [5.6%] compliant). Compared with the most compliant class, the least and moderately compliant classes were younger (mean = 61.9 v. 59.0 v. 53.8 years), had fewer medical conditions per individual (1.37 v. 1.08 v. 0.77), and differed in Hispanic ethnicity (6.2% v. 2.8% v. 9.1%) and COVID-19 vaccine intention (65.8% v. 59.8% v. 35.1%). Compared to the most compliant class, the least compliant class had fewer women (54.6% v. 76.3%), fewer insured individuals (92.2% v. 97.4%), and more withdrew from study participation early (28.6% v. 16.0%). Relative to the most compliant class, the least compliant class had a higher likelihood of COVID-19 infection (OR = 2.08 [95% CI 1.13, 3.85]), lower rate of COVID-19 vaccination (72.6% v. 95.1%), and longer time to 50% COVID-19 vaccination following eligibility (8–9 vs 16 days).

**Conclusions:**

Classes defined by mitigation behaviors compliance had distinct characteristics, including age, sex, medical history, and ethnicity, and were associated with important study and public health outcomes. Targeted public health policies and interventions according to the compliance group characteristics may be of value in current and future pandemic responses to increase compliance.

## Introduction

The coronavirus disease 2019 (COVID-19) pandemic has caused unprecedented public health challenges and loss of life. Between the first COVID-19 case in December 2019 and July 2022, there were more than 551 million cases and 6 million deaths worldwide [1, 2]. To date, there were roughly 3 million cases and 26,000 deaths in North Carolina (NC) [3].

The early pandemic response in NC, guided by recommendations from the Centers for Disease Control and Prevention (CDC), was extensive, including early measures that banned gatherings, closed K-12 public schools and sit-down service in restaurants and bars, and implemented statewide stay-at-home orders, and masking mandates [4–7]. Extensive contract tracing networks were also developed [8]. These mitigation measures controlled the infection rate and incidence of COVID-19 [9–11]. Several studies reported on the use of mitigation behaviors throughout the COVID-19 pandemic in various populations within the United States [12–22]. Most Americans reported using social distancing, sheltering in place, and wearing face coverings [23]. *Hill et al*. examined mitigation behavior habits of North Carolinians [24]. In their study, most individuals wore a mask while at work and away from home and practiced six-feet distancing from members outside of their household at work and while away from home. In other studies, employing COVID-19 mitigation behaviors were associated with an individual’s perceived risk, political voting preference, news source, underlying chronic health conditions, belief in COVID-19-related conspiracy narratives, and demographic characteristics, such as sex, age, education level, race, and ethnicity [25–33]. These studies led us to ask whether unique groups defined by their behaviors could be discerned and whether behavioral groups were associated with important outcomes. If so, understanding the baseline characteristics of these groups might provide a profile that could be used to target education and intervention efforts. Thus, using Latent Class Analysis (LCA), we aimed to create groupings of individuals according to their extent of compliance with COVID-19 mitigation behaviors, describe the key baseline features of the individuals within these groupings, and determine the associations of these behavioral groupings with key study early withdrawal, and pandemic (COVID-19 infection and time to vaccination) outcomes.

## Methods

### Study sample

We used data from 1,410 participants enrolled in the Cabarrus County COVID-19 Prevalence and Immunity (C3PI) Study, a community-based, longitudinal COVID-19 surveillance study. The complete design, methods, and baseline characteristics of the C3PI Study have been published previously [34]. Briefly, C3PI Study participants were recruited from the Measurement to Understand the Reclassification of Disease of Cabarrus/Kannapolis (MURDOCK) Study Community Registry and Biorepository longitudinal cohort study [35, 36]. The study was conducted in Cabarrus County, North Carolina by Duke University. Participants (N = 1,426) were enrolled between June 2020 and August 2020, and completed a baseline survey at enrollment, with biweekly surveys for up to 74 weeks of follow-up (48 weeks in Phase 1 [June 2020 to April 2021] and 26 weeks in Phase 2 [May 2021 to December 2021]). Surveys included questions on the participant’s medical illnesses and medications, symptoms of possible and/or prior COVID-19 illness, testing, and treatment; household features, general health and wellbeing, lifestyle, perceived impact of the pandemic, employment status, perceptions about vaccination, vaccination status, and outlook regarding the pandemic. Additionally, the study recruited a nested weighted, random sample (N = 300) of participants to undergo biweekly SARS-CoV-2 reverse transcriptase-polymerase chain reaction (RT-PCR) testing, and every other month serum sampling for SARS-CoV-2 IgG antibodies during Phase 1 (June 2020-April 2021) and 103 monthly during Phase 2 (May 2021-December 2021). Recruitment into the testing sub-cohort104 followed a weighted, randomized selection schema by sex, age, and race/ethnicity to better reflect the Cabarrus County population distribution.

The current study focused on a set of questions exploring the use of CDC-recommended COVID-19 mitigation behaviors. All participants enrolled into the C3PI Study were considered for this analysis. Participants (N = 29) who did not complete any of the baseline COVID-19 mitigation behavior questions were excluded, leaving a final analysis population of 1,381 (97.9%) individuals.

### Measures

#### Mitigation behaviors

Ten COVID-19 mitigation behaviors were analyzed: 1) wearing a face mask when in public places or at work, 2) washing hands and/or using sanitizer frequently, 3) staying at least 6 feet away from others, 4) avoiding large gatherings, public spaces, or crowds, 5) avoiding contact with high-risk individuals, 6) avoiding food from restaurants including takeout, 7) working or studying from home instead of going into an office or classroom, 8) avoiding shaking hands or touching people, 9) staying home when sick, and 10) wiping down surfaces with disinfectant [37].

### Statistical analysis

#### Latent class analysis

Our primary objective was to estimate classes in the eligible participants at the baseline assessment using LCA models to identify patterns of self-reported levels of COVID-19 mitigation behaviors from an array of the 10 behavior items. LCA statistical testing of model fit and class membership was probabilistic, with latent class membership probabilities computed for each individual from the set of behavior items. Each participant was assigned to a class based on their highest predicted membership probability. Following standard practice, we determined the optimal number of latent classes by specifying a one-class model, adding additional classes sequentially and comparing the Bayesian Information Criterion (BIC) of the candidate models and mean posterior probability estimates of class membership. In addition, we evaluated the resulting models for interpretability [38]. LCA analysis was conducted using SASv9.4 PROC LCA.

#### Latent class membership

In a second step, association of LCA class membership with demographic and baseline characteristics was assessed using categorical analysis methods for categorical measures or analysis of variance for continuous measurements. The “most compliant” group served as the reference group for these assessments.

#### Association of LCA class with outcomes

We modeled the association of LCA compliance class with the following outcomes and modeling described below. Because the LCA compliance class reflects a weighted average summary of the baseline characteristics (thus, is colinear with the group assignment), adjustment for individual baseline characteristics was not conducted.

*Primary outcome*: *Time to vaccination*, calculated as the number of days from the date of eligibility to be vaccinated as determined by the NCDHHS (S1 Table) to the date of vaccination (self-reported). Eligibility criteria were derived from extant MURDOCK Study registry information or the baseline C3PI Study survey instrument. LCA group differences in survival distributions for time to vaccination were evaluated using the Kaplan-Meier method.

*Secondary outcome 1*: *Early withdrawal* (voluntary withdrawal by the participant or administrative withdrawal by study staff if the participant did not respond to four consecutive bi-weekly follow-up surveys), defined as a binary yes/no metric and evaluated using logistic regression, as well as by time to early withdrawal and evaluated using the Kaplan-Meier method.

*Secondary outcome 2*: *COVID-19 infection*, assessed by nasal swab testing via polymerase chain reaction (PCR) in a subsample of the cohort or by participant self-report on a follow-up survey. This outcome was defined as a binary yes/no metric and evaluated using logistic regression.

SAS (Statistical Analysis Software) Version 9.4, SAS Institute Inc, Cary, North Carolina, USA, was used for all analyses. Given the exploratory nature of the analysis, significance for all tests was set at P≤0.05. Exact p-values will be displayed to facilitate interpretation, and no adjustments were made for multiple comparisons.

### Ethics

Both the parent MURDOCK Study (Approval Number: Pro00011196) and Phase 1 and 2 of the C3PI Study (Approval Number: Pro00105703) were approved by the Duke Health Institutional Review Board. Participants provided electronic informed consent within REDCap® to participate in the C3PI Study.

## Results

### Classes of mitigation behavior compliance

**Fig 1:**
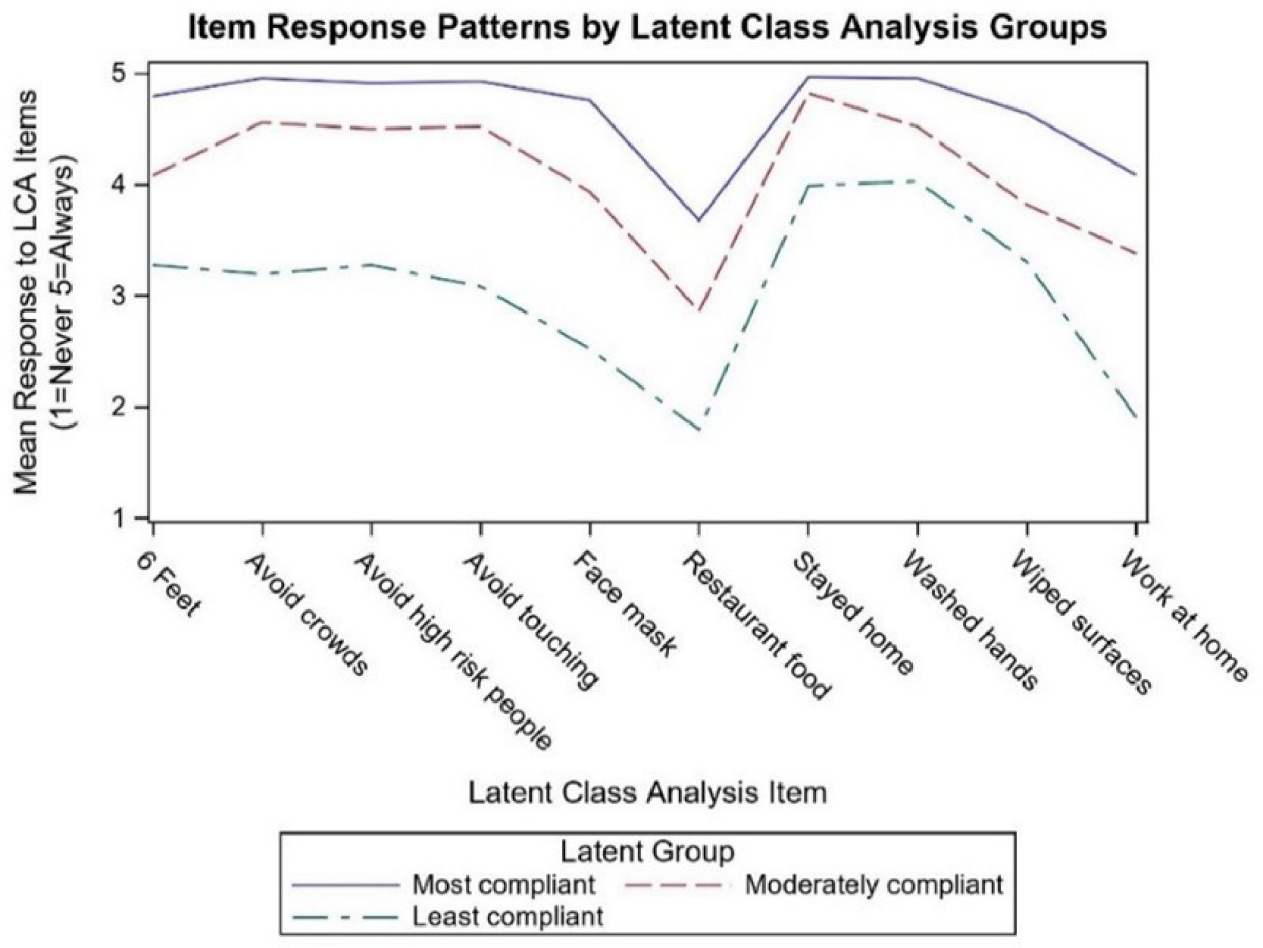
Chosen COVID-19 mitigation behaviors response patterns by latent class analysis class.

A three-class model, depicted in Fig 1, was chosen based on the optimal number of latent classes by comparing the BIC of the candidate models and stopping when the BIC was lowest, thereby indicating the best fit (Table 1). Entropy is a measure of how precisely each LCA solution defines class membership, with higher values indicating higher average probability to a single class. The three-class solution had the lowest BIC value and the highest entropy among the multi-class models (Table 1). The mean posterior probability of individuals correctly assigned to their model class was greater than 0.93 for all classes in the three-class model.

**Table 1:** Summary of model fit criteria.

Number classes	Number (%) in each class				BIC	Adjusted BIC	Entropy
	1	2	3	4			
1	1,381 (100.0)				8978.86	8851.79	1.00
2	771 (55.8)	610 (44.2)			7443.02	7185.72	0.77
3	682 (49.4)	622 (45.0)	77 (5.6)		7202.20	6814.65	0.83
4	369 (26.7)	57 (4.1)	354 (25.6)	601 (43.5)	7332.83	6815.04	0.76

As shown in Fig 1, the classes displayed roughly similar patterns for item response but differed from each other in their mean levels of response. Staying home and washing hands scored high for all three classes, while avoiding restaurant food and working at home were the lowest. The largest class (n = 682, 49.4% of the cohort) ranked highest for mean response score across all ten behavior items processed by LCA, with mean scores higher than 4 (out of 5), suggesting they were “most of the time” or “always” compliant. This class was labeled “most compliant” (class 1). The second most populated class (n = 622, 45.0%) followed a pattern similar to the most compliant class across the ten behaviors but had lower mean scores, meaning they were on average less frequently compliant. This class was labeled “moderately compliant” (class 2). The third class was much smaller (n = 77, 5.6%) and responded on average with lower compliance and was labeled “least compliant” (class 3). The least compliant class differed from the other two classes with much lower mean responses for maintaining six feet difference, avoiding crowds, avoiding high-risk people, avoiding touching others, and using face masks.

### Baseline characteristics

Table 2 shows the characteristics of the overall study population. The cohort was predominately female (69.2%), white (86.2%), overweight (average BMI 28.7 [SD = 6.2]), and well-educated (71.0% college graduate or higher), with an average age of 60.2 (SD = 12.6). Most participants had insurance coverage (96.7%), and over three-quarters had a household income greater than $50,000. Participants had an average of 1.20 (SD = 1.23) chronic medical conditions. Most participants were either employed full-time (42.7%) or retired (35.0%). When asked whether they planned to get the COVID-19 vaccine when it became available, 61.3% responded yes, 30.9% didn’t know, and 7.8% answered no.

**Table 2:** Baseline demographic and clinical characteristics of participants overall and by compliance class.

Characteristic	Overall Sample n = 1,381 (100.0)	Class 1: Most compliant n = 682 (49.4)	Class 2: Moderately compliant n = 622 (45.0)	Class 3: Least compliant n = 77 (5.6)	P-value	
					Class 1 vs. Class 2	Class 1 vs. Class 3
**Female**	955 (69.2)	520 (76.3)	393 (63.2)	42 (54.6)	0.36	0.0005
**Age (Years)**	60.2 (12.6)	61.9 (11.9)	59.0 (13.0)	53.8 (12.9)	< .0001	< .0001
**Race**						
Black/African American	112 (8.1)	71 (10.4)	39 (6.3)	2 (2.6)	0.76	0.18
White	1190 (86.2)	570 (83.6)	553 (88.9)	67 (87.0)		
Other/Missing	79 (5.7)	41 (6.0)	30 (4.8)	8 (10.4)		
**Hispanic Ethnicity**	66 (4.8)	42 (6.2)	17 (2.8)	7 (9.1)	0.001	0.048
**Has Insurance Coverage**	1330 (96.7)	660 (97.4)	599 (96.5)	71 (92.2)	0.41	0.03
**Household Income > $50,000/year**	1030 (78.5)	482 (74.8)	489 (82.5)	59 (77.6)	0.035	0.79
**College Graduate or Higher**	978 (71.0)	470 (69.2)	459 (73.9)	49 (63.6)	0.022	0.13
**BMI**	28.7 (6.2)	28.9 (6.3)	28.4 (6.1)	29.2 (5.9)	0.14	0.70
<25	385 (28.4)	194 (28.9)	173 (28.3)	18 (23.7)	0.59	0.54
25-<30	498 (36.7)	223 (33.2)	246 (40.3)	29 (38.2)		
30+	475 (35.0)	254 (37.9)	192 (31.4)	29 (38.2)		
**Chronic Medical Conditions**						
Chronic Medical Conditions per Participant (out of 14)	1.20 (1.23)	1.37 (1.30)	1.08 (1.16)	0.77 (0.92)	< .0001	< .0001
Allergies	702 (52.6)	355 (54.3)	313 (51.7)	34 (46.0)	0.68	0.24
Asthma	212 (16.2)	113 (17.9)	90 (15.0)	9 (11.8)	0.89	0.30
Diabetes	186 (14.2)	104 (16.4)	74 (12.4)	8 (10.5)	0.77	0.36
Hypertension	460 (34.7)	248 (38.2)	194 (32.2)	18 (24.0)	0.67	0.052
Cardiovascular Disease	116 (8.9)	69 (11.0)	43 (7.2)	4 (5.3)	0.83	0.28
COPD	65 (5.0)	31 (5.0)	31 (5.2)	3 (4.1)	0.68	0.70
Kidney Disease	22 (1.7)	14 (2.2)	7 (1.2)	1 (1.3)	0.79	0.89
Liver Disease	15 (1.2)	6 (1.0)	8 (1.3)	1 (1.3)	0.99	0.89
Cancer	220 (16.8)	119 (18.9)	93 (15.4)	8 (10.5)	0.68	0.14
Immune Disease	102 (7.9)	64 (10.3)	37 (6.2)	1 (1.3)	0.58	0.026
**Substance use**						
Current Tobacco User (Daily Use)	50 (3.6)	21 (3.1)	28 (4.5)	1 (1.3)	0.22	0.34
Current Alcohol User (Weekly Use)	602 (43.7)	276 (40.7)	292 (47.0)	34 (44.2)	0.20	0.95
**Employment Pre-COVID-19**						
Full Time	589 (42.7)	249 (36.5)	297 (47.8)	43 (55.8)	0.47	0.004
Part-Time	127 (9.2)	62 (9.1)	49 (7.9)	16 (20.8)		
Unemployed	16 (1.2)	5 (0.7)	9 (1.5)	2 (2.6)		
Retired	483 (35.0)	264 (38.7)	211 (33.9)	8 (10.4)		
Homemaker	96 (7.0)	53 (7.8)	39 (6.3)	4 (5.2)		
Other	70 (5.1)	49 (7.2)	17 (2.7)	4 (5.2)		
**Work Situation Changed Since Start of COVID-19 Pandemic**	398 (29.3)	200 (30.1)	167 (27.1)	31 (40.3)	0.02	0.03
**How likely will lose job because of COVID-19 (scale 1–10, higher score = more likely)**	2.02 (2.58)	1.96 (2.53)	2.05 (2.57)	2.18 (2.94)	0.55	0.66
**Plan to get vaccine for COVID-19 when it becomes available**						
Yes	839 (61.3)	442 (65.8)	370 (59.8)	27 (35.1)	0.07	< .0001
No	106 (7.8)	45 (6.7)	47 (7.6)	14 (18.2)		
Don’t know	423 (30.9)	185 (27.5)	202 (32.6)	36 (46.8)		
**Received flu vaccine for most recent season (2019–2020)**	1079 (78.8)	550 (81.4)	484 (78.3)	45 (59.2)	0.025	< .0001

Missing values are not included in the denominators. For categorical variables, values represent n (%) within the class. For continuous variables, values represent the mean (SD).

### Mitigation behaviors compliance class characteristics

Table 2 shows the sociodemographic characteristics by compliance class. Compared with the most compliant class, the proportion of women was lower in the least compliant group (54.6% v. 76.3%, p = 0.0005), and participants in the moderately compliant and least compliant classes were younger (mean age 61.9 vs. 53.8 vs 59.0 years, both comparisons p < .0001). The distribution of race did not vary by compliance class. The proportion of Hispanic participants was lower in the moderately compliant (2.8%, p = 0.001) and higher in least compliant (9.1%, p = 0.048) classes compared with the most compliant class (6.2%). Insurance coverage was high across all three classes but significantly lower in the least compliant compared with the most compliant class (97.4% vs. 92.2%, p = 0.03). Both the moderately compliant and least compliant classes had fewer average numbers of medical conditions per participant (moderately compliant [1.08] and least compliant [0.77]) compared with the most compliant class (1.37; both comparisons p<0.0001).

Distribution of pre-COVID employment categories differed between the most and least compliant classes (p = 0.004). The least compliant class had more full-time (55.8% vs. 36.5%) and part-time (20.8% vs. 9.1%) employed individuals and fewer retired individuals (10.4% vs. 35.0%) compared with the most compliant class.

In response to COVID-19 vaccine intention, the least compliant class had a lower proportion of Yes responses (35.1% vs. 65.8%) and a higher percentage of No (18.2% vs. 6.7%) and Don’t Know (46.8% vs. 27.5%) responses than the most compliant group. Compared with the most compliant class (81.4%), the least compliant and moderately compliant classes had lower percentages of individuals who received a flu vaccine for the most recent season (moderately compliant [78.3%, p = 0.025] and least compliant [59.2%, p < .0001]).

### Relationship of mitigation behaviors compliance class to specific outcomes

#### Primary outcome: Vaccination

**Fig 2:**
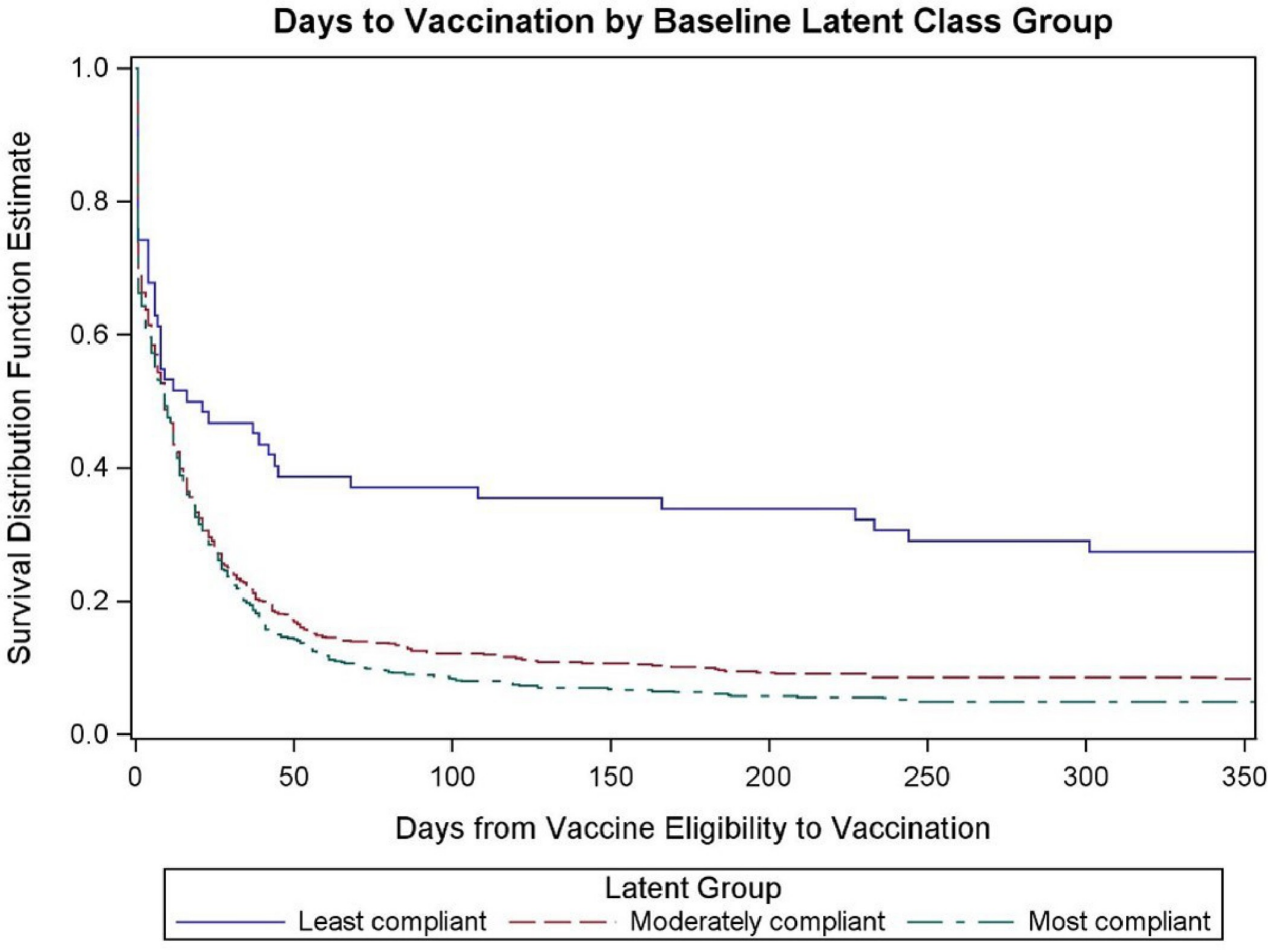
Days to vaccination by compliance class.

At the end of the study, the most compliant class had the highest percentage of individuals who received a COVID-19 vaccine (95.1%), followed by the moderately compliant (91.7%) and the least compliant (72.6%). Fig 2 displays the survival estimates using each day as the time increment with an outcome event of receiving a COVID-19 vaccine. The most compliant and moderately compliant classes achieved 50.0% COVID-19 vaccination between days eight and nine following eligibility, while the least compliant class reached the 50.0% vaccination milestone on day 16 from eligibility. Around day 50, the vaccination rate slowed in all groups, with the least compliant class ending with 28.0% unvaccinated compared with <10.0% unvaccinated in the other 2 classes. Using the log-rank test, time to vaccination was similar for the moderately compliant class compared with the most compliant group (p = 0.19), but was significantly longer for the least compliant class compared with the most compliant group (p<0.0001).

#### Secondary outcome #1: Study withdrawal

Overall, 220 (15.9%) participants withdrew from the study before study completion. The least compliant class (28.6%) had the highest percentage of early withdrawal, followed by the most compliant (16.0%) and the moderately compliant (14.3%) classes. Compared with the most compliant class, the moderately compliant and least compliant classes had odds ratios (OR) for early withdrawal of 0.88 (95% CI 0.65–1.19) and 2.10 (95% CI 1.23, 3.59), respectively. Expressed as the number of days to early termination from the baseline survey date, compared with the most compliant class, the moderately compliant and least compliant classes had hazard ratios (HR) for early withdrawal of 1.13 (95% CI 0.85–1.51) and 1.73 (95% CI 1.09, 2.75), respectively.

#### Secondary outcome #2: Infection

There were 154 COVID-19 infections overall (11.2%). The infection rate in the least compliant class (19.5%) was higher than either the moderately compliant (10.9%) or the most compliant (10.4%) class. Compared with the most compliant class, the odds of COVID-19 infection in the moderately compliant class were 1.06 (95% CI 0.74–1.50) and 2.08 (95% CI 1.13, 3.85) in the least compliant class.

## Discussion

Using latent class analysis, we identified three classes of mitigation behaviors compliance (most compliant: 682/1,381 [49.4%]; moderately complaint: 622/1,381 [45.0%]; least compliant: 77/1,381 [5.6%]) that had distinct class membership characteristics and that were associated with key COVID-19 pandemic outcomes (time to vaccination, COVID-19 infection) and study outcomes (early withdrawal). Time to 50% vaccination from first eligibility was twice as long in the least compliant compared with the most compliant class and the proportion not getting vaccinated by the end of the study period was nearly 3-fold higher. Importantly, the odds of COVID-19 infection was over twice as great in the least compliant class as the most compliant class (2.08 [95% CI 1.13, 3.85]). These findings provide important insights for consideration in public health approaches to current and future pandemic education and management by providing a profile of those least compliant with mitigation behaviors for public health officials to use to focus their outreach efforts.

LCA has been used to study class membership relative to mitigation behaviors compliance in other studies. Smail et al. used responses to a Health Belief Model to create a 3 class model to assess the likelihood of associations between class membership, health beliefs, and sociodemographic characteristics [25]. They found differences in class membership linked to age category, sex, racial/ethnic group, education level, and census region. In another study, mitigation behaviors class in a 4-class model was associated with confidence in government, empathy, internal locus of control, age, risk-taking behaviors, trait openness, self-isolation patterns, conscientiousness, and change in mental health status [39]. Similar associations between compliance class and participant protective measure beliefs, social attitudes, personality, level of worry about COVID-19, COVID-19 information consumption, and attitudes towards government and other cultural factors were found in study defining a 2 class model [40]. Finally, in a study of 157 German participants, compliance patterns in a 3 class model were associated with age, gender, and aspects of public stigma [41]. However, none of these studies employing LCA explored associations between class membership and study outcomes or COVID-19 pandemic outcomes.

### Strengths and limitations

In our longitudinal cohort study with detailed baseline and subsequent biweekly follow-up surveys, we obtained a considerable amount of behavioral, clinical, and sociodemographic information as well as key study (early withdrawal) and pandemic (COVID-19 infection and time to vaccination) outcomes on a large cohort (N = 1,381). However, the study population was regionally based, had limited diversity (69% women and 86% white race) and was highly educated, which may limit generalizability. Hence, the estimates presented could overfit the model, and attenuate the findings in more diverse populations. Replication in more diverse cohorts would be important before broad implementation outside the studied region.

While all surveys were administered frequently (baseline, followed by biweekly follow-up surveys), participant responses could be subject to biases, such as recall bias, and opinions at baseline were likely to change during the course of the pandemic. All LCA classes experienced early withdrawal; however, the least compliant class experienced the highest proportion of early withdrawal (28.6%). The differential early withdrawal rates by LCA class may have resulted in limited power in the least compliant class and introduced some unknown amount of bias into the estimates of the outcomes assessed during follow up. However, the point estimates provided are good indicators of places for future research. An important reason to employ LCA analyses is to find distinct groups, where small groups may be indicative of a very important group to research.

The process to create the LCA groups is an exploratory data-driven process; the number of groups that result is unknown a priori. It was anticipated that LCA group profiles would be defined not only by the items included in the LCA process (in this case the 10 mitigation behavior items), but also by differential associations with demographic and baseline characteristics. Thus, the LCA class represents a weighted average summary of these characteristics. As such, considering the demographic and baseline characteristics as confounders with adjustment for them would have the effect to drive the LCA group associations with the outcomes towards the null. Therefore, we believe the optimal interpretation of the LCA group associations with the outcomes is the unadjusted analysis.

### Implications for the future

Because mitigation behaviors compliance classes were associated with important pandemic (infection and vaccination) and study (early withdrawal) outcomes, delineating the clinical, sociodemographic, and belief characteristics of the groups has implications for future pandemic management strategies (e.g., education, interventions, community reach) and research study retention efforts. Compared with the most compliant class, the least compliant class was younger and had more men, Hispanic individuals, and part-time and full-time workers, while having less insurance coverage, fewer medical conditions per person and retired individuals, and less flu vaccinations for the most recent season. By understanding the profile of individuals in the least compliant class, health policy makers and researchers may be able to better target messaging strategies and interventions in the current and future pandemics.

## Conclusion

Groups defined by their compliance with COVID-19 mitigation behaviors had distinct clinical and sociodemographic characteristics and perspectives on vaccination. Groupings by compliance with COVID-19 mitigation behaviors were associated with important study (early withdrawal) and public health (COVID-19 infection and time to vaccination) outcomes. Public health policies and interventions directed according to behavioral class characteristics defined using LCA may be of value in current and future pandemic responses.

## Supporting information

S1 Table North Carolina vaccine availability dates by priority group. (DOCX)
