# Supplementary material for: Mitigation behavior prior to COVID-19 vaccination availability is associated with COVID-19 infection and time to vaccination
Source: PLoS One. 2023 Mar 24;18(3):e0283381. doi: 10.1371/journal.pone.0283381 (PMC10038251; doi:10.1371/journal.pone.0283381)
Supplement: S1 Table — (DOCX) [file pone.0283381.s001.docx]

**S1 Table. North Carolina Vaccine Availability Dates by Priority Group.**

| **Group Number** | **Group** | **Vaccine Availability Date** | **Source** |
| --- | --- | --- | --- |
| 1 | Health Care Workers and Long-Term Care Staff and Residents | December 14, 2020 | 1 |
| 2 | Older Adults | January 18, 2021 | 2 |
| 3 | Frontline Essential Workers - child care and PreK-12 school workers | February 24, 2021 | 3 |
|  | Additional frontline essential workers | March 3, 2021 | 4 |
| 4 | People with high-risk medical conditions who have not been vaccinated | March 17, 2021 | 5-7 |
|  | People living in a close group living setting |  |  |
|  | Other essential workers not yet vaccinated | March 31, 2021 |  |
|  | Other people in close group living settings |  |  |
| 5 | All Adults | April 7, 2021 | 8 |
| 6 | Teens | May 12, 2021 | 9,10 |
| 7 | Children | November 2, 2021 | 11 |

**References**

1. NCDHHS. Deeper Dive Group 1 – Health Care Workers and Long-Term Care Staff and Residents. Accessed December, 2021. <https://covid19.ncdhhs.gov/media/1120/open>

2. NCDHHS. Deeper Dive Group 2 – Older Adults. Accessed December, 2021. <https://covid19.ncdhhs.gov/media/1121/open#:~:text=Adults%2065%20and%20older%20are,is%20greater%20than%20vaccine%20supply>.

3. NCDHHS. Deeper Dive Group 3 – Frontline Essential Workers (School and Child Care). Accessed December, 2021. <https://covid19.ncdhhs.gov/media/1417/open>

4. NCDHHS. Deeper Dive Group 3 – Frontline Essential Workers. Accessed December, 2021. <https://covid19.ncdhhs.gov/media/1148/open>

5. NCDHHS. Deeper Dive Group 4 – Adults at High Risk for Exposure and Increased Risk of Severe Illness. Accessed December, 2021. <https://covid19.ncdhhs.gov/media/1171/open>

6. Gov. Cooper Announces People with High-Risk Medical Condition in Group 4 Will Be Eligible for COVID-19 Vaccine Beginning March 17. March 11, 2021. Accessed December 2021. <https://governor.nc.gov/news/press-releases/2021/03/11/gov-cooper-announces-people-high-risk-medical-condition-group-4-will-be-eligible-covid-19-vaccine>

7. Governor Cooper Announces Accelerated Timeline for Vaccination Eligibility. March 25, 2021. Accessed December 2021. <https://governor.nc.gov/news/press-releases/2021/03/25/governor-cooper-announces-accelerated-timeline-vaccination-eligibility>

8. North Carolina’s COVID-19 Vaccine Eligibility Opens for All Adults on April 7. April 6, 2021. <https://governor.nc.gov/news/press-releases/2021/04/06/north-carolinas-covid-19-vaccine-eligibility-opens-all-adults-april-7#:~:text=Page%20Program-,North%20Carolina's%20COVID%2D19%20Vaccine%20Eligibility%20Opens,All%20Adults%20on%20April%207>

9. FDA and CDC Authorize Pfizer COVID-19 Vaccine for Children Age 12 and Older. May 13, 2021. Accessed December 2021. <https://www.ncdhhs.gov/news/press-releases/fda-and-cdc-authorize-pfizer-covid-19-vaccine-children-age-12-and-older>

10. Whitehead B. CDC endorses Pfizer shot for 12-year-olds; NC opens appointments for kids 12-15. Updated May 12. Accessed December, 2021. <https://wlos.com/news/local/cdc-endorses-pfizer-covid-19-shot-for-children-12-and-up-north-carolina-opens-appointments-for-kids-age-12-15>

11. NCDHHS. COVID-19 Vaccine Update. Updated August 3, 2022. Accessed December, 2021. <https://covid19.ncdhhs.gov/media/905/open>
